# Evaluation of the Effectiveness of a Problem-Solving Intervention Addressing Barriers to Cardiovascular Disease Prevention Behaviors in 3 Underserved Populations: Colorado, North Carolina, West Virginia, 2009

**DOI:** 10.5888/pcd11.130249

**Published:** 2014-03-06

**Authors:** Christa L. Lilly, Lucinda L. Bryant, MSHA, Janie M. Leary, Maihan B. Vu, Felicia Hill-Briggs, Carmen D. Samuel-Hodge, Colleen R. McMilin, Thomas C. Keyserling

**Affiliations:** Author Affiliations: Lucinda L. Bryant, Colleen R, McMilin, University of Colorado Anschutz Medical Campus, Aurora, Colorado; Janie M. Leary, Fairmont State University School of Education, Health, and Human Performance, Fairmont, West Virginia; Maihan B. Vu, Carmen D. Samuel-Hodge, Thomas C. Keyserling, The University of North Carolina at Chapel Hill, Chapel Hill, North Carolina; Felicia Hill-Briggs, Johns Hopkins University, Baltimore, Maryland.

## Abstract

**Introduction:**

In low-income and underserved populations, financial hardship and multiple competing roles and responsibilities lead to difficulties in lifestyle change for cardiovascular disease (CVD) prevention. To improve CVD prevention behaviors, we adapted, pilot-tested, and evaluated a problem-solving intervention designed to address barriers to lifestyle change.

**Methods:**

The sample consisted of 81 participants from 3 underserved populations, including 28 Hispanic or non-Hispanic white women in a western community (site 1), 31 African-American women in a semirural southern community (site 2), and 22 adults in an Appalachian community (site 3). Incorporating focus group findings, we assessed a standardized intervention involving 6-to-8 week group sessions devoted to problem-solving in the fall of 2009.

**Results:**

Most sessions were attended by 76.5% of participants, demonstrating participant adoption and engagement. The intervention resulted in significant improvement in problem-solving skills (P < .001) and perceived stress (P < .05). Diet, physical activity, and weight remained stable, although 72% of individuals reported maintenance or increase in daily fruit and vegetable intake, and 67% reported maintenance or increase in daily physical activity.

**Conclusion:**

Study results suggest the intervention was acceptable to rural, underserved populations and effective in training them in problem-solving skills and stress management for CVD risk reduction.

## Introduction

Cardiovascular disease (CVD), including coronary heart disease and stroke, continue to be the leading causes of adult mortality and morbidity in the United States (1). Over 80 million American adults have 1 or more types of CVD (2), hypertension being the most common. Although healthy behaviors such as weight control, physical activity, good nutrition, and smoking cessation can prevent or decrease the negative effects of CVD, behavior change to reduce CVD risk is difficult to accomplish and to maintain and this may be especially so for hard-to-reach populations who are often at highest risk (3).

The Centers for Disease Control and Prevention’s Heart Disease and Stroke Prevention Division created the Prevention Research Center’s Cardiovascular Health Intervention Research and Translation Network (PRC CHIRTN) to develop and implement a coordinated applied research and translation agenda and to conduct prevention research and translation activities to promote cardiovascular health (CVH) (4). In 2007–2008 PRC CHIRTN conducted a qualitative study, CVH Perceptions, to increase understanding of knowledge and perceptions of risks, disease characteristics, and prevention of CVD, with a focus on underserved and understudied populations. Findings identified barriers to lifestyle changes that would promote CVH in PRC CHIRTN’s diverse underserved partner communities. Stressful life circumstances among adults with multiple life roles (eg, employee, parent, caregiver for older family members and others) emerged as a common barrier across populations studied (5).

Although structural factors not easily amenable to change (eg, financial situation, geographic location, discrimination) contribute to stressful circumstances, behavioral interventions such as problem-solving and stress management (6,7) can help people address the barrier of stressful circumstances so that they can be more successful at improving lifestyle behaviors. Problem-solving is a long-standing behavior change intervention approach that teaches skills for managing life stressors and barriers that impede optimal physical and emotional functioning (8). Problem-solving therapy has been used successfully as a patient behavioral self-management intervention for cancer (9), weight loss (10), diabetes (11), and several other symptoms and conditions (7).

By using CVH Perceptions findings and incorporating the studied communities’ preferences and concerns, 3 of the PRC CHIRTN centers (University of North Carolina–Chapel Hill [UNC-CH], West Virginia University [WVU], University of Colorado Anschutz Medical Campus [UC-AMC]) collaborated to create a CVD intervention for people living in the context of stressful life circumstances. This CVD intervention is an adaptation of an existing problem-solving program for self-management of diabetes and CVD (12). The study reported here assessed the development and pilot implementation of the adapted CVD intervention. Given the formative nature of the study, the feasibility and acceptability of the intervention were the primary outcomes.

## Methods

### Development of the intervention

Responding to findings from CVH Perceptions, building from a theoretical framework, and returning to the targeted communities to gain community-specific perspectives, we developed a problem-solving intervention, Decide 2 Care for You, to promote heart-healthy behaviors by adults with stressful life circumstances. Project DECIDE (12,13), which was based on social problem-solving theory (8,14), was the basis for Decide 2 Care for You. Project DECIDE was a didactic program that provided education in problem solving, coaching, modeling, rehearsal and practice, performance feedback, shaping, and positive reinforcement for skill development (8,12). For lifestyle counseling, we used materials from the New Leaf program (15); these materials were designed for use in clinical settings serving low-income populations (15–17) and support individually tailored lifestyle counseling (18).

Each site (WVU, UNC-CH and UC-AMC) obtained institutional review board approval from its respective university for each aspect of the study (need's assessment and evaluation and implementation of the intervention).

### Community input

At the 3 sites, investigators collected information and later implemented the program in different populations and settings. Focus groups (site 1: 2 groups, 18 participants; site 2: 4 groups, 17 participants; site 3: 2 groups, 16 participants) provided insights about how to create an acceptable program and market it to the local community in the summer of 2009. Specifically, site 1 (UNC-CH) included older African American women attending an African American church located in a semirural area of central North Carolina. Site 2 (WVU) included multigenerational adults from an Appalachian community center. Site 3 (UC-AMC) included Hispanic and non-Hispanic white adult women from worksites in the rural San Luis Valley of southern Colorado. Common themes included the following:

- Desired topics: healthy weight, healthy behaviors
- Preferred activities and structure: hands-on demonstrations; active learning relevant to entire family; support systems; a workout buddy; fun, engaging, social; accurate information
- Interventionist, group leader: a “real person” who has been through the process, overcome similar obstacles, experienced some health issues, and can tell his or her story with humor, and enthusiasm, not necessarily a health professional but someone with experience and the knowledge base
- Facilitators: incentives, food, worksite location, seeing results
- Barriers: time more than money
- Recruitment: churches, stores and businesses, newspapers, word-of-mouth

### Decide 2 Care for You intervention

Building from the Project DECIDE curriculum (19) and incorporating focus group input, we designed Decide 2 Care for You (http://hpdp.unc.edu/research/projects/chirtn/) as an 8-session program. Each site’s IRB also approved the evaluation and implementation of the intervention study. Because of scheduling constraints, site 2 combined sessions 2 and 3 and sessions 4 and 5; site 3 combined Sessions 6 and 7, and site 1 scheduled the sessions for every 1 to 2 weeks. Sessions were offered twice during the week; however, site 1 and site 3 sessions lasted approximately 2 hours, while site 2 sessions lasted approximately 90 minutes. To train interventionists from each of the communities, an expert conducted a 1-day training session. Once the intervention began, weekly telephone conferences with the interventionists were held to promote sharing successful techniques, resolving problems, and maintaining fidelity.

### Sample and recruitment

Each site created a sampling frame suitable for its target population. The recruitment goal was 2 groups of 15 adults at each site (a total of 90 participants across the 3 study sites). Study staff contacted potential participants by telephone to provide information about the study. Participants at all sites received $20 for the data collection sessions (cash, gift cards, or checks depending on university policy) and $5 for each of the remaining program sessions. All participants gave signed, informed consent. The intervention took place at all sites during the fall of 2009.

### Measures and data collection

Participants completed baseline measures, including a demographic form, during an enrollment session before the first intervention session. Responses to questions adapted from the Physical Activity Readiness Questionnaire (20) determined participants’ ability to engage safely in moderate-intensity physical activity; a “yes” response to any of the questions excluded the participant from the physical activity component of the intervention. Throughout the data collection procedures, the interventionists were available as necessary to read questionnaire items or answer any questions the participants had. Materials were reviewed for language and terminology appropriateness by the investigators and interventionists before intervention implementation. For more information about the materials and measures used, see http://hpdp.unc.edu/research/projects/chirtn/.

Study measures assessed at the baseline enrollment visit and final study visit included the following 5 variables:

1. Weight without shoes: average of 2 measurements using an electronic scale, measured at each of the intervention visits.
2. Problem-solving skills: assessed with the 50-item Health Problem-Solving Scale (HPSS) (19) including questions such as “I know that my choices about taking care of my health condition(s) make a difference in how things come out in the end,” with responses on a 5-point Likert-type response scale (1= not at all true of me, 2 = a little true of me, 3 = moderately true of me, 4 = very true of me, and 5= extremely true of me). Higher means indicated more capable problem-solving skills. Internal reliability with this sample was excellent, Cronbach’s α = 0.95.
3. Stress: assessed by 14 items (21) (with this sample, Cronbach’s α = 0.83), which included items such as “In the last month, how often have you been upset because of something that happened unexpectedly?” on a 5-point Likert-type response scale (1 = never, 2 = almost never, 3 = sometimes, 4 = fairly often, and 5 = very often). Higher means indicated more perceived stress.
4. Fruit and vegetable intake: assessed with 10 items (22) and averaged for a daily serving score.
5. Physical activity: assessed by answers to the following question: “Thinking about all different kinds of physical activity, including on the job, in the yard, or planned exercise, how many minutes do you usually spend each day doing activities that really get you moving (make you breathe harder)?”

In addition, a 25-question acceptability questionnaire developed for this study was administered at the final visit. Of the original 25 questions, 11 questions examined acceptability and feasibility of materials and activities, group leader and group format, participant interaction, usefulness of the problem-solving approach, and program structure, with a consistent 4-point Likert-type response (1 = not at all satisfied, 2 = not very satisfied, 3 = somewhat satisfied, and 4 = very satisfied).

### Analysis

Statistical analyses were conducted using SPSS, version 18.0 (IBM Corp, Chicago, Illinois) and SAS, version 9.2 (SAS Corp, Cary, North Carolina). Three responses to physical activity questions had extreme values and were excluded. Analyses included descriptive statistics and repeated measures of ANOVAs to assess main effects of time, group, and the interaction between time and group for continuous pre- and postvariables; significant omnibus tests were analyzed with Tukey pair-wise comparisons by group. Main effects for time were examined because weight were taken pre-, post- and at every session whereas the other outcomes were taken pre- and postintervention. Matched pair *t* tests were conducted for within-site differences of pre- and post-results. Results were considered significant at α = 0.05. Last known weight was brought forward for missing weight values; weight results were examined with regard to attendance. Acceptability and feasibility questions were examined by using frequencies; because of the low numbers of negative responses, the 2 negative-response categories were combined for some items.

## Results

Groups differed on demographic variables because of inherent differences in the participants recruited at each site (Table 1). Attendance averaged 76.5% across all sites and sessions. Of the initial 81 participants enrolled, 8 (10%) dropped out within the first 2 sessions. The majority of participants missed 2 or fewer sessions (61; 75%), and 29 participants (36%) attended all sessions. Reasons for not being able to attend were recorded for 62% of missing sessions and included illness (13.6%), family conflict or obligations (12.9%), work demands (11.4%), being out of town (6%), and transportation problems (4.6%). Sixty-eight participants (84%) returned for the final intervention/data collection visit. Of the 68 participants at the last session, 22 attended at site 1 (75.9%), 21 at site 2 (95.5%), and 25 at site 3 (80.6%).

**Table 1:** Participant Characteristics, Problem-Solving Intervention Addressing Barriers to Cardiovascular Disease Prevention Behaviors at Sites in 3 States: North Carolina, West Virginia, Colorado, 2009.

Characteristic^a^	Study Overall (N = 81)	Site 1^b^ (N = 28)	Site 2^c^ (N = 31)	Site 3^d^ (N = 22)
**Age, y, mean (SD)**	52.8 (12.2)	60.1 (90.1)	51.6 (13.2)	45.9 (9.8)
**Sex**				
Female	74 (90.2)	28 (100.0)	23 (74.2)	22 (100.0)
**Education**				
Less than high school	5 (6.4)	2 (7.4)	2 (6.4)	1 (4.8)
High school/GED	25 (32.1)	6 (22.2)	14 (46.8)	5 (23.8)
Some college	19 (24.3)	8 (29.6)	6 (19.9)	5 (23.8)
College graduate/post-graduate	29 (37.2)	11 (40.8)	8 (26.9)	10 (47.6)
**Race/ethnicity**				
Hispanic	15 (19.0)	0	1 (3.4)	14 (63.6)
Black/African American	29 (35.8)	28 (100.0)	1 (3.2)	0
Non-Hispanic white	44 (54.3)	0	30 (96.8)	14 (63.6)
Other/not indicated	5 (22.7)	0	0	5 (22.7)
**Employment**				
Full-time	52 (64.2)	10 (35.7)	20 (64.5)	22 (100.0)
Part-time	6 (7.4)	3 (10.7)	2 (6.5)	1 (4.5)
Unemployed/retired	20 (24.7)	15 (53.5)	5 (16.1)	0 (4.5)
**Household composition**				
1 adult (self)	24 (29.6)	13 (46.4)	6 (19.4)	5 (22.7)
2+ adults	57 (70.4)	15 (53.6)	25 (80.6)	17 (77.3)
Any children	40 (50.6)	10 (35.7)	11 (37.9)	19 (86.4)
**Health insurance**				
Currently has health insurance	71 (88.8)	28 (100.0)	25 (80.6)	18 (85.7)
**Cardiovascular health status**				
Diagnosed with high blood pressure	40 (49.4)	24 (85.7)	12 (38.7)	4 (18.2)
Diagnosed with high cholesterol	36 (45.0)	11 (39.3)	18 (60.0)	7 (31.8)
Diagnosed with diabetes	15 (18.8)	6 (21.4)	6 (19.4)	3 (14.3)
Ever smoked	5 (6.2)	2 (7.1)	3 (9.7)	0
**Diet experience of past 2 years**				
Tried weight loss diet program	39 (49.4)	8 (28.6)	18 (62.1)	13 (59.1)
Currently following a diet program	8 (10.0)	2 (7.1)	3 (10.0)	3 (13.6)

a Data presented as frequency and percentages unless otherwise designated.

b University of North Carolina at Chapel Hill.

c West Virginia University.

d University of Colorado Anschutz Medical Campus.

Five outcomes of interest were compared: perceived problem solving, perceived stress, daily fruit and vegetable intake, weekly minutes of physical activity, and weight (Table 2).

**Table 2:** Participant Problem Solving, Perceived Stress, and Selected Lifestyle Outcomes at Baseline (N = 81) and Follow-up (N = 68), Problem-Solving Intervention Addressing Barriers to Cardiovascular Disease Prevention Behaviors, at sites in 3 states: North Carolina, West Virginia, Colorado, 2009.

Outcome	Group^a^	N	Preintervention (Baseline)		Postintervention (Follow-up)		*P* -Values^b^
			Mean (SD)	Range	Mean (SD)	Range	
Problem solving scale^c^	Total	68	135.2 (26.0)	64–196	146.7 (26.8)	73–192	<.001
	Site 1	22	154.3 (24.0)	111–196	154.5 (29.1)	73–191	.97
	Site 2	25	129.1(28.5)	72–188	146.6 (23.8)	108–188	.001
	Site 3	21	122.1(25.6)	64–167	138.8 (27.4)	81–192	.002
Perceived stress scale^d^	Total	68	37.9 (6.8)	21–56	36.0 (7.7)	19–56	<.05
	Site 1	22	34.9 (6.2)	23–45	33.3 (7.5)	19–51	.19
	Site 2	25	40.0 (8.2)	22–56	37.7 (8.1)	22–56	.14
	Site 3	21	38.9 (6.0)	21–49	37.1 (7.5)	22–53	.16
Fruit and vegetable servings per day	Total	67	4.2 (2.0)	0–12	4.7 (1.7)	0.7–9	.057
	Site 1	21	4.9 (2.2)	2–12	4.9 (1.5)	2–8	1.00
	Site 2	25	4.1 (2.4)	0–10	4.9(2.2)	0–9	.08
	Site 3	21	3.6 (1.3)	2–6	4.3(1.3)	1–6	.057
	Total	42	37.4 (23.0)	0–120	37.9 (27.3)	0–180	.91
Physical activity (min per day)^e^	Site 1	11	54.6 (29.0)	15–120	36.4 (22.7)	15–90	.13
	Site 2	17	37.1 (22.2)	5–90	46.2 (36.1)	0–180	.32
	Site 3	14	20.4 (17.9)	0–120	31.1 (23.2)	0–80	.15
	Total	81	204.91 (50.0)	113–354	204.4 (50.3)	107–359	.31
Weight^f^	Site 1	28	205.84 (39.5)	133–267	204.6 (38.1)	130–262	.29
	Site 2	31	208.58 (50.3)	125–351	209 (51.2)	126–359	.51
	Site 3	22	198.57 (61.9)	113–354	197.8 (62.7)	107–357	.29

Abbreviation: CI, confidence interval.

a Site 1, University of North Carolina at Chapel Hill; site 2, West Virginia University; site 3, University of Colorado Anschutz Medial Campus.

b *P*-values reported for the matched *t* test within site for each site, and the time effect of the ANOVA for the total.

c Higher scores for problem-solving scales indicate improved skills.

d Higher scores for perceived stress scale indicate more stress.

e Three participants’ data for physical activity were removed from analysis due to extreme values.

f For missing weight values, the last weight was brought forward.

### Problem solving

Problem-solving skills (PSS) improved significantly, from mean 135.2 to mean 146.7 for the total study group (*F* [1, 64] = 18.04, *P* < .001). Each group increased from baseline except site 1. Pair-wise comparisons indicated that site 1 participants had significantly higher baseline PSS than did site 3 (*P* < .01). Site 2’s baseline PSS scores were not significantly different from those of either site 1 or site 3. These differences could be attributed to site 1’s initially high scores, which were maintained at follow-up. Within-site analysis indicated that both site 2 and site 3 showed significant increases from pre- to postintervention (*P* < .002), whereas site 1 maintained a high mean from baseline to follow-up.

### Perceived stress

Perceived stress (PS) decreased significantly across the whole study group from baseline (37.9) to follow-up (36.0) (*F* [1, 64] = 6.05, *P* < .05). Differences existed between groups at baseline and follow-up (*P* < .05); pair-wise comparisons indicated that site 1 had significantly lower scores than site 2 (mean difference = −4.73, *P* < .05); site 3 PS scores did not differ significantly from either those of site 1 or site 2 at baseline or follow-up.

### Fruits and vegetables

There were no significant differences from baseline to follow-up (*P* = .057) or between the groups (*P* = .171) for number of servings of fruits and vegetables although number of servings increased, albeit not significantly, for site 2 (from 4.1 to 4.9 servings, *P* =.08) and site 3 (from 3.6 to 4.3, *P* = .06), whereas site 1 maintained an initially high baseline number of servings at follow-up (4.9). Individually, 43 (64%) participants reported any increase in daily fruit and vegetable intake, and 48 (72%) maintained or increased their daily fruit and vegetable intake.

### Physical activity

There was no significant difference in number of minutes of physical activity from baseline to follow-up (*P* = 0.913) in the total study group. However, a significant difference was found between groups (*F* [2, 39] = 3.41, *P* < .05), although no differences were apparent in post-hoc pair-wise comparisons. Within sites, both site 2 and site 3 increased in minutes per day (site 1, from 37.1 to 46.2 minutes, *P* = .32; site 3 from 20.4 to 31.1, *P* = .15) (not significant increases), whereas the minutes for site 1 decreased from a mean of 54.6 minutes per day to 36.4. Individually, 20 participants (44%) reported some increase in daily minutes of physical activity, and 30 (67%) maintained or increased their physical activity.

### Weight

Although weight change was not a primary outcome, it was measured at every session as part of the intervention program. The mean weight of the entire study group remained virtually the same across time (204.9 to 204.4 pounds) and within sites (Table 2). An examination of attendance by weight revealed that the 20 participants who attended fewer than 75% of the sessions were on average heavier than the 61 participants with better attendance (218.4 pounds vs 199.9, *P* = .15), although this difference was not significant.

### Participant satisfaction

Table 3 presents selected acceptability questionnaire results from program completers (84%, N=68). Nearly all (91.2%) participants reported that they used their newly acquired problem-solving skills to address a problem or problems. Most participants (70.6%) reported that they learned a lot about the problem-solving process. The majority of participants (57.4%) found problem-solving very useful, and 41.2% found it somewhat useful. Most participants (70.6%), however, reported that they were only somewhat successful in solving the problems.

**Table 3:** Participant Responses (N = 68) to Select Acceptability and Feasibility Questions, Problem-Solving Intervention Addressing Barriers to Cardiovascular Disease Prevention Behaviors in 3 Underserved Samples, at sites in 3 states: North Carolina, West Virginia, Colorado, 2009.

Acceptability and Feasibility Questions	Positive response^a^, N (%)	Slightly positive response^b^, N (%)	Negative response^c^, N (%)
Thinking about the Decide 2 Care for You program overall, how satisfied were you with what the program offered?	47 (69.1)	18 (26.5)	3 (4.4)
Thinking about the Decide Program overall, how satisfied were you with the Group leader?	59 (86.8)	8 (11.8)	1 (2.9)
Thinking about the Decide Program overall, how satisfied were you with how group sessions went?	47 (69.1)	19 (27.9)	2 (2.9)
Thinking about the Decide Program overall, how satisfied were you with the way participants interacted with each other?	56 (82.4)	10 (14.7)	2 (2.9)
I learned a lot about the problem solving process in the group sessions.	48 (70.6)	15 (22.1)	5 (7.4)
How useful did you find the focus on problem solving as an approach to improve diet and physical activity?	39 (57.4)	28 (41.2)	1 (1.5)
How successful have you been in solving your problems?	14 (20.6)	48 (70.6)	6 (8.8)
How easy to understand was the information you heard during the group?	60 (88.2)	7 (10.3)	1 (1.5)
How important was it for you to work with the group leader to set specific goals to improve your eating habits and physical activity habits?	34 (52.3)	25 (38.5)	6 (9.2)
How satisfied were you with the amount of information and help the group leader gave you about eating habits?	52 (77.6)	14 (20.9)	1 (1.5)
How satisfied were you with the amount of information and help the group leader gave you about being more physically active?	51 (76.1)	16 (23.9)	0

a Positive responses were “strongly agree,” “very useful,” and “very successful.”

b Slightly positive responses were “agree,” “somewhat useful,” and “somewhat successful.”

c Negative responses were “strongly disagree,” “disagree” “not at all successful,” “not at all useful,” “not very successful” and “not very useful.”

In terms of the materials, participants found the following information very useful: healthful eating (88.1%), the New Leaf materials (82.1%), and physical activity (73.1%). The problem-solving workbook, the facts and information booklet, physical activity and relaxation stretches, and information about problem-solving were also found very useful by at least 70% of participants. Log sheets, the food provided, and information about stress reduction were found very useful by 49% to 63% of participants. Fewer than 10% of participants found these materials not at all or not very useful.

Participants were asked to name the top 3 aspects of the Decide 2 Care for You Program that were helpful in making lifestyle changes. Among participants 4 aspects were given as the top 3: 1) problem-solving workbook, 2) New Leaf materials, 3) weekly weight checks, and 4) group format. Other areas of the Decide 2 Care for You Program, including satisfaction with the group leader, the way in which participants interacted with each other, and ease of understanding presented information, were viewed overwhelmingly as positive.

In terms of program procedures, most participants (61.2%) thought the number of sessions was “just right,” and 31.3% thought there were “not enough” sessions. At site 3 (8 sessions long) most participants (45% of that group) said there were not enough sessions; site 2, however, had fewer sessions (7 sessions total), and only 24% of that group responded that there were not enough sessions. Across groups, 83.8% thought the length of each session was just right. Only at site 2 (with 90-minute-long sessions) did a few participants (16%) report that the sessions were too short. Across groups, 46.3% of participants thought that the program should last 6 or more months instead of 2 to 3 months, and 57.4% would be willing to attend the longer program. Perhaps because at least 2 different sessions were offered for each group, only 13.2% of participants had to regularly take time off from work to attend. By the time the program ended, 87.7% of participants had already shared the information they learned with others.

## Discussion

The goal of this project was to implement a CVD intervention, Decide 2 Care for You, for 3 underserved populations. The intervention was designed to teach problem-solving skills relevant to improving lifestyle behaviors known to affect CVD risk in the context of stressful life circumstances. Results of focus group discussions before intervention implementation informed the acceptability, marketing, content, context, and environment of the intervention. Themes from the focus groups informed our adaptation of an established 8-session patient problem-solving and training curriculum, Project DECIDE, to reduce CVD risk. Results of the Decide 2 Care for You intervention suggest important and clinically relevant improvements not only to the participants’ problem-solving skills but also to their behaviors related to nutrition and stress.

Site-specific differences suggest that demographic characteristics of the study participants may play some role in the effectiveness of the intervention. It may be important to target this intervention to people who have the greatest risk-reduction needs.

Most participants attended most sessions, diagnosed a problem or barrier to a healthful lifestyle, learned about problem solving, and established a good start in overcoming that particular problem. Supplemental information about healthful eating and physical activity, incorporated from the New Leaf program, were found to be particularly helpful for all groups. Flexibility in offering sessions was important for working adults, and the group format and leader were vital to the program’s success. We recommend that future implementation of this adapted intervention (Decide 2 Care for You program) include supplemental material such as New Leaf and other practical healthy lifestyle materials, a variety of session times and locations, a group format, and using a group leader who has qualities desired by focus group participants. In response to suggestions that more sessions might be helpful, we recommend that the program be given over a longer time frame.

This study has limitations. Given the pre–post design without a control group and the fact that many reported outcomes are self-reported behaviors, reported improvement must be interpreted cautiously. In addition, the sample was small, and though all come from settings where stressful life circumstances are common, our findings may not be generalizable to other samples with high prevalence rates of stressful circumstances. Although we believe the additional information given by varying delivery between sites was useful, this may have also resulted in varying program assessment results between sites.

This study suggests problem-solving interventions of modest duration are both acceptable and feasible for adult populations at high risk for stressful life circumstances and hold promise as an effective behavioral approach to promote CVH for high-risk populations. Future research should focus on tailoring this type of intervention to the specific needs of the target population, expanding the duration to ensure that behavior change is maintained over time, and testing the effectiveness with appropriately designed randomized trials.
